# Etymologia: *M. bovis*

**DOI:** 10.3201/eid2103.ET2103

**Published:** 2015-03

**Keywords:** etymologia, Mycobacterium bovis, M. bovis, bacteria, tuberculosis and other mycobacteria, bacillus Calmette-Guérin vaccine, BCG, Robert Koch, Theobald Smith, Albert Calmette, Camille Guérin

## M. bovis

From the Latin *bos* (“ox” or “cow”) *Mycobacterium bovis* is a virulent bacterial species originally isolated from tubercules in cattle. Robert Koch, who discovered the tubercle bacillus in 1882, believed that *M. bovis* was not a danger to humans. Theobald Smith and others established beyond doubt that, contrary to Koch’s belief, *M. bovis* could infect humans but was not the usual source of human infection. In 1908, French scientists Albert Calmette and Camille Guérin chose an *M. bovis* strain for their work on a tuberculosis vaccine. They repeatedly subcultured the isolate on a mixture of glycerol, potato, and bile for 13 years until it was sufficiently attenuated to be used as a vaccine. The bacillus Calmette-Guérin (BCG) vaccine was adopted by the League of Nations as the standard tuberculosis vaccine in 1928 and continues to be used in most developing countries.
